# Comparative study and meta-analysis of meta-analysis studies for the correlation of genomic markers with early cancer detection

**DOI:** 10.1186/1479-7364-7-14

**Published:** 2013-06-05

**Authors:** Zoi Lanara, Efstathia Giannopoulou, Marta Fullen, Evangelos Kostantinopoulos, Jean-Christophe Nebel, Haralabos P Kalofonos, George P Patrinos, Cristiana Pavlidis

**Affiliations:** 1 Faculty of Mathematical, Physical and Natural Sciences, Department of Biological Sciences, University of Trieste, Trieste, 34128, Italy; 2 School of Health Sciences, Department of Pharmacy, University of Patras, University Campus, Rio, Patras 26504, Greece; 3 Clinical Oncology Laboratory, Division of Oncology, Department of Medicine, University of Patras, Rio, Patras, 26504, Greece; 4 School of Computing and Information Systems, Faculty of Science, Engineering and Computing, Kingston University, London, SW15 3DW, UK

**Keywords:** Cancer, Meta-analysis, Gene, Association, Interaction, Single-nucleotide polymorphism, Alleles, Clustering

## Abstract

A large number of common disorders, including cancer, have complex genetic traits, with multiple genetic and environmental components contributing to susceptibility. A literature search revealed that even among several meta-analyses, there were ambiguous results and conclusions. In the current study, we conducted a thorough meta-analysis gathering the published meta-analysis studies previously reported to correlate any random effect or predictive value of genome variations in certain genes for various types of cancer. The overall analysis was initially aimed to result in associations (1) among genes which when mutated lead to different types of cancer (e.g. common metabolic pathways) and (2) between groups of genes and types of cancer. We have meta-analysed 150 meta-analysis articles which included 4,474 studies, 2,452,510 cases and 3,091,626 controls (5,544,136 individuals in total) including various racial groups and other population groups (native Americans, Latinos, Aborigines, etc.). Our results were not only consistent with previously published literature but also depicted novel correlations of genes with new cancer types. Our analysis revealed a total of 17 gene-disease pairs that are affected and generated gene/disease clusters, many of which proved to be independent of the criteria used, which suggests that these clusters are biologically meaningful.

## Introduction

Cancer is the result of a complicated process that involves the accumulation of both genetic and epigenetic alterations in various genes [1]. The somatic genetic alterations in cancer include point mutations, small insertion/deletion events, translocations, copy number changes and loss of heterozygosity [2]. These changes either augment the action and/or expression of an oncoprotein or silence tumour suppressor genes. Single-nucleotide polymorphism (SNP) is the most common form of genetic variation in the human genome. Although common SNPs for disease prediction are not ready for widespread use [3], recent genome-wide association studies (GWASs) using high-throughput techniques have identified regions of the genome that contain SNPs with alleles that are associated with increased risk for cancer such as *FGFR2* in breast cancer [4-7].

The knowledge on gene mutations that predispose tumour initiation or tumour development and progress will give an advantage in cancer patients' treatment. Despite the complexity and variability of cancer genome, numerous studies have examined the correlation of genome variation with cancer development and progression [8]. However, ambiguous results have been generated from the attempt to link genome variants with cancer prediction or detection. A literature search revealed that even among several meta-analyses, there were unclear results and conclusions.

We have, therefore, conducted a thorough meta-analysis of meta-analysis studies previously reported to correlate the random effect or predictive value of genome variations in certain genes for various types of cancer. The aim of the overall analysis was the detection of correlations (1) among genes whose mutation might lead to different types of cancer (e.g. common metabolic pathways) and (2) between groups of genes and types of cancer.

## Methods

We performed a thorough field synopsis by studying published meta-analysis studies involving the association of various types of cancer with SNPs located in certain genomic regions. For each published meta-analysis included in our study, we also investigated the number of patients (cases) and controls, date, type of study, study group details (e.g. gender, race, age, etc.), measures included, allele and genotype frequency and also the outcome of each study, i.e. if there was an association or not, the interactions noticed in each of these studies, etc.

We have meta-analysed 150 meta-analysis articles (Additional file 1), which included 4,474 studies, 2,452,510 cases and 3,091,626 controls (5,544,136 individuals in total). The meta-analyses that have been meta-analysed included various racial groups, e.g. Caucasians, Far Eastern populations (Asian, Chinese, Japanese, Korean, etc.), African-American and other population groups (native Americans, Latinos, Aborigines, etc.). Three types of studies were included: (1) pooled analysis, (2) GWAS and (2) other studies, e.g. search in published reports. Collected data consisted of a list of genes, genomic variants and diseases with a known genotype-phenotype association (whether or not a given variation has an impact on susceptibility to a given disease). The principle of our study was to use data mining techniques to find groups (referred to as clusters hereafter) of genes or diseases that behave similarly according to related data. Such groupings will make it possible to find different cancer types susceptible to similar genotypes as well as different genes associated to similar cancer types. Furthermore, our approach would facilitate predicting whether susceptibility to one type of cancer may be indicative of predisposition to another cancer type. Moreover, the association between a group of genes and a given phenotype may suggest that these genes interact or belong to the same biochemical pathway. In order to allow data mining analysis, genotype-phenotype associations had to be classified within a fixed set of categories, i.e. yes/small yes/may/no. Moreover, genes or diseases with fewer than two entries were not considered in our analysis since their clustering would not be meaningful.

Then, data were processed using a state-of-the-art general purpose clustering tool, CLUTO [9]. Data analysis consisted in finding the tightest and most reliable groupings. Since CLUTO offers a wide range of methods, and many different scoring schemes can be used to estimate similarity between genotypes or phenotypes, cluster reliability was assessed by their robustness to clustering criteria (details are provided in Additional file 1). As a consequence, each putative association has been qualified as either ‘highly consistent’ or ‘moderately consistent’. The biological significance of those clusters was, first, evaluated using the Search Tool for the Retrieval of Interacting Genes/Proteins (STRING) [10,11], a biological database and web resource of known and predicted protein-protein interactions. The STRING database contains information from numerous sources, including experimental data, computational prediction methods and public text collections. It is widely accessible, and it is regularly updated. Second, literature research was performed to complete this initial evaluation.

## Results and discussion

In this study, we performed a meta-analysis of published meta-analysis studies to investigate possible correlations among genes and SNPs and various types of cancer, as well as among gene-gene and/or gene-environmental interactions. Furthermore, an advanced literature research was applied in order to evaluate our results obtained from our meta-analysis. Our data were not only consistent with previously published literature but we have also depicted novel correlations of genes with new types of cancer. Our analysis showed a total of ten cancer-related genes that are affected (Table 1).

**Table 1:** Summary of genes and SNPs identified by meta-analysis to be positively correlated with various cancers

**Gene**	**Cancer type**	**SNPs**		**References**	**Supporting references**
		**rs number**	**Other name**		
*ERCC2*	BC	rs13181	p.K715Q	[12,13]	[14-17]
*ERCC2*	BC	rs1799793	p.D312N	[12,18]	[14-16]
*ERCC2*	LC	rs13181	p.K751Q	[19,20]	[17,21,22]
*ERCC2*	LC	rs1799793	p.D312N	[23]	[17,21,22]
*CCND1*	BC	rs603965	c.870G>A	[24]	[25-31]
*CYP2E1*	CRC	rs3813867	NA	[32]	[32-41]^a^
*CYP2E1*	HNC	rs3813867	NA	[42,43]	[44]
*CYP2E1*	HNC	rs6413432	NA	[42]	[44]
*GSTP1*	CRC	rs1695	p.I105V	[45]	[39,46-55]
*IL6*	BC	rs1800795	c.-174G>C	[56,57]	
*MTHFR*	GC	rs1801131	c.1298A>C	[58]	[59,60]^b^
*MTHFR*	BC	rs1801131	c.677C>T, c.1298A>C	[61,62]	[63,64]
*SOD2*	BC	rs4880	p.V16A, p.A9V	[62,65,66]	
*TGFB1*	BC	rs1800469	NA	[67-69]	
*TGFB1*	BC	rs1800470	NA	[67,70-73]	
*TGFB1*	BC	rs1982073	NA	[74]	[64,75-77]
*TP53*	BC	rs1042522	p.R72P	[78,79]	[80-94]
*TP53*	UBC	rs1042522	p.R72P	[95]	[96-100]
*TP53*	CRC	rs1042522	p.R72P	[78,101-103]	[104-108]
*TP53*	CRC	rs17878362	NA	[78]	[104-108]
*TP53*	EC	rs1042522	p.R72P	[109,110]	[111]
*TP53*	LC	rs1042522	p.R72P	[78]	[112-117]
*TP53*	LC	rs17878362	NA	[78]	[112-117]
*VEGFA*	BC	rs3025039, rs699947	c.936C>T, c.-2578C>A	[20,45,118,119]	[120]

These findings are supported by the published literature. ^a^For a different SNP (rs1329149); ^b^for c.677C>T and c.1298A>C. *NA* not available.

### Correlation of SNPs' genes with various types of cancer

The association highlighted by our meta-analysis between the *CYP2E1* gene and colorectal cancer (CRC), head and neck cancer (HNC) and liver cell carcinoma (LLC) is supported by published data [33-39,44,121]. An additional literature search to evaluate our initial results revealed novel correlations of the gene combination *CYP2E1* and *GSTM1* with prostate cancer (PC) susceptibility, lung cancer (LC) and bladder cancer (UBC) as shown in Table 2[126-128]. A similar correlation was found in CRC using a knockdown model [32,40,41]. Studies not only confirm the possibility of association between the *CCND1* gene and breast cancer (BC) [25] but also suggest involvement with squamous cell carcinoma (SCC), oesophageal cancer (EC), oral cancer (OC) and malignant glioma (MG), as arisen from the interaction between the *CCND1* and *CCND3* genes [26,122-124]. This is further corroborated in mouse model studies that show association of *CCND1* with BC [25,27-31,153] and PC [125].

**Table 2:** Summary of genes and SNPs identified by further literature search as positively correlated with various cancers

**Gene**	**Cancer type**	**SNPs**		**References**
		**rs number**	**Other name**	
*CCND1*	OC	rs603965	c.870G>A	[26,122-124]
*CCND1*	PC	rs603965	c.870G>A	[125]
*CYP2E1*	PC	NA	NA	[126]
*CYP2E1*	LC	NA	NA	[127]
*CYP2E1*	UBC	NA	NA	[128]
*CYP2E1*	OC	NA	NA	[40]
*ERCC2*	OC	rs1799793, rs13181	p.D312N, p.K751Q	[23]
*ERCC2*	HNC	rs1799793, rs13181	p.D312N, p.K751Q	[129-131]
*GSTP1*	PC	rs1695	p.I105V	[126,128,132,133]
*MTHFR*	BCC	rs1801131	c.677C>T, c.1298A>C	[134]
*MTHFR*	ALL	rs1801131	c.677C>T, c.1298A>C	[59,135,136]
*MTHFR*	LC	rs1801131	c.677C>T, c.1298A>C	[137]
*MTHFR*	UBC	rs1801131	c.677C>T, c.1298A>C	[138]
*MTHFR*	CC	rs1801131	c.677C>T, c.1298A>C	[139]
*MTHFR*	NHL	rs1801131	c.677C>T, c.1298A>C	[140,141]
*MTHFR*	HNC	rs1801131	c.677C>T, c.1298A>C	[142]
*TGFB1*	GC	rs1982073	c.+29C>T	[143]
*TGFB1*	LC	rs1982073	c.+29C>T	[144]
*TGFB1*	PC	rs1982073	c.+29C>T	[145]
*TGFB1*	PC	rs1982073	c.+29C>T	[146]
*TGFB1*	CRC	rs1982073	c.+29C>T	[147]
*TP53*	EmCa	rs1042522/rs17878362	p.R72P	[148]
*TP53*	PC	rs1042522/rs17878362	p.R72P	[114,149]
*TP53*	OVCa	rs1042522/rs17878362	p.R72P	[150]
*TP53*	GC	rs1042522/rs17878362	p.R72P	[151]
*TP53*	OC	rs1042522/rs17878362	p.R72P	[152]

*NA *not available.

Moreover, as far as the *ERCC2* is concerned along with the association of *ERCC1* gene with BC and LC which is already confirmed [14-17,21,22], we have also identified from our further literature search on humans the existence of an association with OC [26] and with HNC [129-131]. There were no similar mouse studies that could confirm or overrule our findings.

Our findings regarding the *GSTP1* gene are confirmed by the published literature [39,46-55]. Furthermore, we have noticed an association with PC derived from the combination of *GSTM1* and *CYP1A1*[126,128,132,133]. Likewise, previous experimental evidence supports the association we found between the *MTHFR* gene and BC, basal cell carcinoma (BCC) [63,134] and gastric cancer (GC) [59,60]. An association was also found between *MTHFR* gene with other types of cancer, such as acute lymphoblastic leukaemia (ALL) [135,136,154], LC [137], UBC coming from interaction between *CTH* and *GSTM1*[138], CRC [139], non-Hodgkin's lymphoma (NHL) [140,141], BC [64] and HNC [142]. Specifically, in the case of NHL, the gene combination of *MTHFR* and *TYMS* might influence the susceptibility to *NHL*[140,141].

Concerning *TGFB1*, apart from the BC [64] that was confirmed from the results of our further literature search on humans and on mouse model [75,76], we have noticed also the following associations with gastric dysplasia, LC, pancreatic cancer (PanC) and BC [77,143-146]. Also, an association of *TGFB1* with CRC was found using a mouse model [147].

In addition for *TP53* gene, we have observed in the results of our meta-analysis that it is associated with BC, UBC, CRC, EC and LC [80-87,96-100,104-108,111-113,149]. We have observed also that *TP53* gene might be associated with OC [88,148], too. Concerning the literature research on knockout mice, we have confirmed the associations with BC [89-94] and LC [114-117], and we have found also associations with ovarian cancer (OVCa) [150], GC [151] and OC [152]. Moreover for the *VEGFA* gene, based on further literature *TGFB1* research, we have confirmed the association with BC [120], but we had not found any other evidence supporting the association with other types of cancer.

### Correlations between groups of genes and various types of cancer

We have examined and confirmed the highly consistent gene clustering results over further literature search via STRING. Our search revealed additional types of cancer, except from the types that we have studied in our meta-analysis that seems to be related with pair of genes. STRING database reports binding interaction between *GSTP1* and *GSTM1* genes, activating interaction between *MMP2* and *EGF* genes, between *VEGFA* and *IL1B* genes and between *MMP-9* and *IL8* genes (Table 3). The application of our machine learning method has highlighted that those pair of genes have similar association profiles and, therefore, might be involved in the same pathways. The genes that do not appear in the associations do not probably correlate with the presence of a certain type of cancer.

**Table 3:** Putative gene-gene associations with various cancer types

**Gene associations**		**Considered phenotypes**	**Comments**	**STRING confirmation**	**Literature confirmation**
**Gene 1**	**Gene 2**				
*GSTP1*	*GSTM1*	4		Binding interaction	[Reference]: study type
*TGFB1*	*IL6*	5	4 of 5 based on ‘yes’		
*MMP2*	*EGF*	3	Based on ‘yes’	Activating interaction	
*VEGFA*	*IL1B*	2		Activating interaction	
*MMP9*	*IL8*	4	Based on ‘may’	Activating interaction	KEGG: same process
*MMP1*	*MMP3*	5	Based on ‘may’		

First, in our meta-analyses, we observed that the interaction between *IL6* and *TGFB1* genes was associated to the following types of cancer: BC, CRC, GC, LC and PC as shown in Table 4. Although further literature search on humans could not validate our highly consistent results, we discovered that these interactions are associated to additional types of cancer, such as HNC [187], CRC [158], renal cancer (RC), small cell lung cancer [188], malignant melanoma (MM) [189-192] and OVCa [193]. Additionally, regarding our further research on the interaction between *IL6* and *TGFB1* genes on mouse models, we have confirmed our initial results principally for BC [155-157] and PC [159] and have noticed associations with epithelial cancer [194], skin tumour [195], LC [196], OVCa and cervical cancer (CC) [197,198] and HNSCC [199]. Second, we found that the interaction between *MMP-2* and *EGF* was associated with LC, BC and GC (Table 4). Subsequently with a further literature search, we confirmed the association with BC osteolysis [163,164] and also found new associations with EC [200], LC, RC and PC [162]. Furthermore, in some cases, we have observed the association of the aforementioned genes with OSCC [201]. In this study, *EGF* induced *MMP-1* expression that is required for type I collagen degradation. In addition, *MMP-1* is also associated with human papillomavirus [202] and BC [165].

**Table 4:** Summary of gene-gene interactions and the corresponding SNPs in these genes

**Gene 1**	**Gene 2**	**Cancer type**	**SNP's gene 1**		**SNP's gene 2**		**References (gene 1)**	**References (gene 2)**	**Supporting references**
			**rs number**	**Other name**	**rs number**	**Other name**			
*IL6*	*TGFB1*	BC	rs1800795	c.-174G>C	rs1800469, rs1800470	c.-509C>T, p.T29C	[56]	[67-70,72-74]	[155-157]
*IL6*	*TGFB1*	CRC	rs1800795	c.-174G>C	rs1800470	p.T29C	[57]	[71]	[158]
*IL6*	*TGFB1*	GC	rs1800795	c.-174G>C	rs1800470	p.T29C	[57]	[71]	
*IL6*	*TGFB1*	LC	rs1800795	c.-174G>C	rs1800470	p.T29C	[57]	[71]	
*IL6*	*TGFB1*	PC	rs1800795	c.-174G>C	rs1800470	p.T29C	[57]	[71]	[159]
*MMP2*	*EGF*	LC	rs2438650	c.-1306C>T	rs4444903	c.61A>G	[160]	[161]	[162]
*MMP2*	*EGF*	BC	rs2438650	c.-1306C>T	rs4444903	c.61A>G	[160]	[161]	[163-165]
*MMP2*	*EGF*	GC	rs2438650	c.-1306C>T	rs4444903	c.61A>G	[160]	[161]	
*VEGFA*	*IL1B*	BC	rs3025039	c.936C>T	rs114327	NA	[166-169]	[170]	[171]
*VEGFA*	*IL1B*	BC	rs699947	c.-2578C>A	rs1143634	NA	[172]	[170]	[171]
*VEGFA*	*IL1B*	BC	NA	NA	rs16944	NA	NA	[170]	[171]
*VEGFA*	*IL1B*	GC	rs3025039	c.936C>T	rs3087258	NA	[45]	[173]	
*VEGFA*	*IL1B*	GC	rs699947	c.-2578C>A	NA	IL1B-31-ami	[95]	[173]	
*MMP9*	*IL8*	BC	rs3918242	c.-1562C>T	rs4073	c.-251A>T	[160]	[174]	[171]
*MMP9*	*IL8*	CRC	rs3918242	c.-1562C>T	rs4073	c.-251A>T	[160]	[174]	
*MMP9*	*IL8*	GC	rs3918242	c.-1562C>T	rs4073	c.-251A>T	[160]	[175]	
*MMP9*	*IL8*	LC	rs3918242	c.-1562C>T	rs4073	c.-251A>T	[160]	[174]	
*MMP1*	*MMP3*	BC	rs1799750	c.-1607 1G>2G	rs3025058	c.-1171 5A>6A	[176]	[176]	
*MMP1*	*MMP3*	CRC	rs1799750	c.-1607 1G>2G	rs3025058	c.-1171 5A>6A	[176]	[176]	
*MMP1*	*MMP3*	HNC	rs1799750	c.-1607 1G>2G	rs3025058	c.-1171 5A>6A	[176]	[176]	
*MMP1*	*MMP3*	LC	rs1799750	c.-1607 1G>2G	rs3025058	c.-1171 5A>6A	[176]	[176]	[177,178]
*MMP1*	*MMP3*	OVCa	rs1799750	c.-1607 1G>2G	rs3025058	c.-1171 5A>6A	[176]	[176]	
*GSTP1*	*GSTM1*	CRC	rs1695	p.I105V	rs1065411	GSTM1 present/null	[45]	[179]	
*GSTP1*	*GSTM1*	BC	rs1695	p.I105V	rs1065412	GSTM1 present/null	[180]	[181]	[182,183]
*GSTP1*	*GSTM1*	OVCa	rs1695	p.I105V	rs1065413	GSTM1 present/null	[184]	[184]	
*GSTP1*	*GSTM1*	UBC	rs1695	p.I105V	rs1065414	GSTM1 present/null	[185]	[186]	

These were identified in our meta-analysis. Their correlation with various cancer types is also shown. *NA *not available.

Another interesting interaction that was revealed from our analysis was between the *VEGFA* and *IL1B* genes that were associated with BC and GC (Table 4). After proceeding with a further literature search, we have not found similar results - except from one report [171] - but we have identified additional associations with HNC, ALL, laryngeal carcinoma and MM [203-206]. For *MMP-9* and *IL8* interaction, there was no study confirming our initial results for BC, CRC and GC on neither humans nor mouse models. We have observed though that there was evidence for an association with nasopharyngeal carcinoma [171], LC [177,178] and UBC [207]. Similarly, we could not find any study that could support the interactions between *MMP-1* and *MMP-3* and *GSTP1* with *GSTM1*, although two studies confirmed that *GSTP1* and *GSTM1* interactions could be associated with BC [182,183] (Table 4).

Indications from further literature search on human models revealed associations for *MMP-1* and *MMP-3* with types of cancer such as BCC, metatypical cancer of the skin [208], colorectal adenoma and RC [209,210], and for *GSTP1* and *GSTM1*, endometrial cancer (EmCa) [211], LC [212], multiple myeloma (observed no significant association to prostatic adenoma and adenocarcinoma) [213], PC [133,214], ALL [215], chronic myeloid leukaemia [216] and PanC [217].

**Figure 1:**
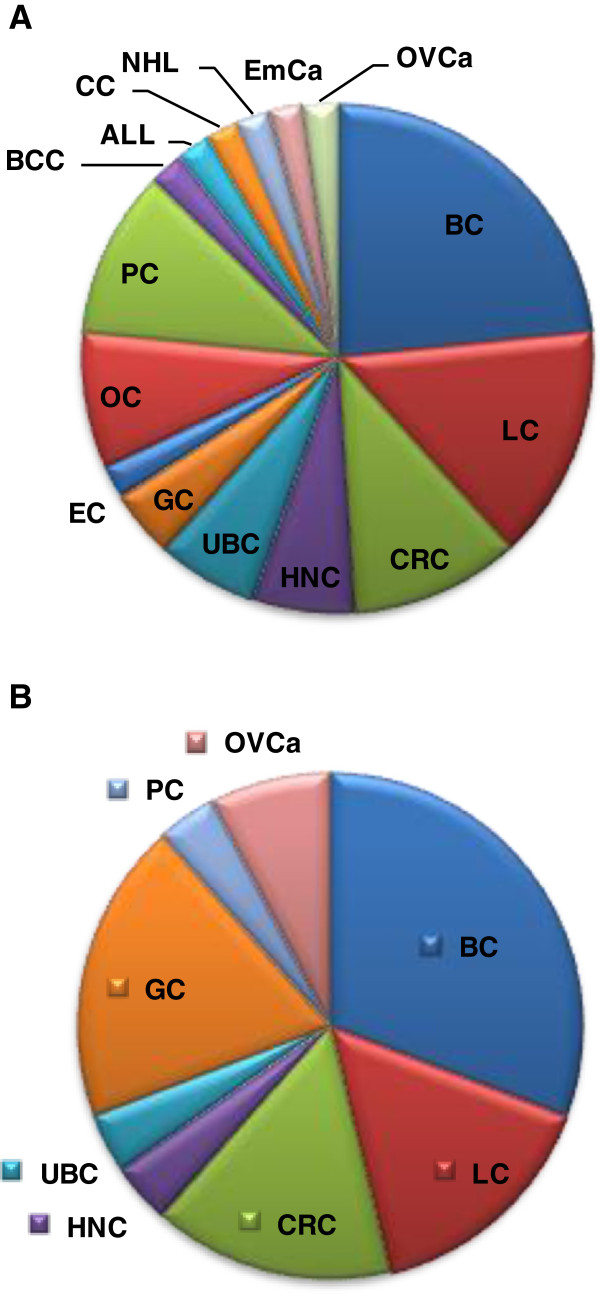
**The distribution of various cancer types. **According to (**A**) the number of SNPs per cancer type and (**B**) the number of genes or gene correlations per cancer type. By extrapolating the data in Tables 1, 2, 3 and 4, it seems that the number of genome variations and genes is profoundly bigger in BC, probably indicating that this type of cancer is not a single disease but, most likely, a spectrum of related disease states.

We have then attempted to depict the various types of cancers according to the number of SNPs and genes and/or gene clusters found from our meta-analysis to be meaningfully associated with certain cancer types. Our data indicate that BC is correlated more often than the other types of cancer both with the number of SNPs (Figure 1A) as well as with the number of genes or gene clusters (Figure 1B). This observation underlies the heterogeneity of BC, indicating that it is, most likely, not a single disease but a spectrum of related disease states.

## Conclusions

In essence, our meta-analysis study generated clusters of genes and diseases, many of which proved to be independent of the criteria used, which suggests that these clusters are most likely biologically meaningful. Preliminary study of some clusters and of our results shows that indeed these genes interact. As regards the associations, with a further literature analysis on human and mouse models, we have also found meaningful gene associations related to other cancer types not previously reported in the literature, an observation that warrants further investigation.

## Competing interests

The authors declare that they have no competing interests.

## Authors' contributions

ZL carried out the data collection, result analysis and participated in the manuscript preparation. EG participated in the manuscript preparation and data analysis. MF participated in the result and statistical analysis and manuscript revision. EK participated in the data collection and manuscript revision. JCN carried out the result and statistical analysis and participated in the manuscript preparation. HPK participated in the manuscript preparation. GPP participated in the design of the study, data analysis and manuscript preparation. CP conceived of the study, participated in its design and coordination as well as manuscript preparation. All authors read and approved for the final manuscript.

## Supplementary Material

Additional file 1 Genes and cancer types included in this meta-analysis. Click here for file
