# Supplementary material for: Comparative study and meta-analysis of meta-analysis studies for the correlation of genomic markers with early cancer detection
Source: Hum Genomics. 2013 Jun 5;7(1):14. doi: 10.1186/1479-7364-7-14 (PMC3686617; doi:10.1186/1479-7364-7-14)
Supplement: Additional file 1 — Genes and cancer types included in this meta-analysis. [file 1479-7364-7-14-S1.docx]

**Additional file 1**

# Comparative study and meta-analysis of meta-analysis studies for the association of genomic markers with early cancer detection

Zoi Lanara ^1,2^, Efstathia Giannopoulou ^3^, Marta Fullen ^4^, Evangelos Kostantinopoulos ^2^, Jean-Christophe Nebel ^4^, Haralabos P. Kalofonos ^3^, George P. Patrinos ^2^, Cristiana Pavlidis ^2,#^

^1^ University of Trieste, Faculty of Mathematical, Physical and Natural Sciences, Depatment of Biological sciences, Italy

^2^ University of Patras, School of Health Sciences, Department of Pharmacy, Patras, Greece

^3^ Clinical Oncology Laboratory, Division Of Oncology, Department of Medicine, University of Patras, Patras, Rio, Greece, 26504

^4^ Kingston University, School of Computing and Information Systems, Faculty of Science, Engineering and Computing, London, UK

We have conducted a meta-analysis of meta-analyses studies that include the following genes and cancer types:

I.1. Genes

- *ABCB1*
- *ADH1B*
- *ALDH2*
- *APE1*
- *ATM*
- *AURKA*
- *CAG*
- *CASP8*
- *CCND1*
- *CHRNA3*
- *CHRNA5*
- *COMT*
- *COX2*
- *CTLA4*
- *CYP17*
- *CYP19*
- *CYP1A1*
- *CYP1A2*
- *CYP1B1*
- *CYP2E1*
- *EGF*
- *ELAC2*
- *Enos*
- *ERCC2*
- *ERCC2/XPD*
- *ESR2*
- *FGFR2*
- *GPX1*
- *GSTA1*
- *GSTM1*
- *GSTP1*
- *GSTT1*
- *has miR 146*
- *has miR 196*
- *HER2*
- *HIF- 1A*
- *HNF1B*
- *Hogg1*
- *HSD17B1*
- *IFN-γ*
- *IGFBP3*
- *IL-10*
- *IL-1B*
- *IL-1RN*
- *IL-6*
- *IL8*
- *IL-1β*
- *LSP1*
- *MAP3K1*
- *MDM2*
- *MGMT*
- *MMP-1*
- *MMP-2*
- *MMP-3*
- *MMP7*
- *MMP9*
- *MnSOD*
- *MPO*
- *MTHFR*
- *MTR*
- *MTRR*
- *MUTYH*
- *MYC*
- *NAT2*
- *NBS1*
- *NOD2*
- *NOS3*
- *NQO1*
- *P21*
- *p53*
- *PgR*
- *PPARγ*
- *RAD51*
- *SOD2*
- *SRD5a2*
- *SULT1A1*
- *TGFB1*
- *TNFα*
- *TNRC9*
- *TP53*
- *TP63*
- *UGT1A1*
- *VEGF*
- *VEGFA*
- *XPD*
- *XRCC1*
- *XRCC2*
- *XRCC3*

I.2. Genomic Loci

- *12q13.13*
- *12q13.3*
- *1q41*
- *20q13.33*
- *3q26.2*
- *9p24*

II. Cancer types:

- AC: Adenocarcinoma
- ALL: Acute Lymphoblastic Leukemia
- BC: Breast cancer
- BCC: Basal cell canrcinoma
- CC: Cervical cancer
- CML: Chronic Myeloid Leukemia
- CRC: Colorectal cancer
- CRCad: Colorectal adenoma
- EC: Esophageal cancer
- EmCa: Endometrial cancer
- EPCa: Epithelial cancer
- GC: Gastric cancer
- HNC: Head and neck cancer
- LaC: Laryngeal cancer
- LC: Lung cancer
- LC: Liver Cancer
- MCS: Metatypic carcinoma of the skin
- MG: Maglignant glioma
- MM: Malignant melanoma
- NPC: Nasopharyngeal carcinoma
- NHL: non Hodkins lymphoma
- OC: Oral cancer
- OVCa: Ovarian cancer
- PanC: Pancreatic cancer
- PC: Prostate cancer
- RC: Renal cancer
- SC: Skin cancer
- SCC: Squamous cell carcinoma
- SCCLC: Small-Cell Lung Cancer
- ST: Skin Tumor
- UBC: Bladder cancer
